# The Institute of Medicine’s call to action revisited: assuring access to public health education for U.S. college students

**DOI:** 10.3389/fpubh.2023.1185845

**Published:** 2023-04-27

**Authors:** Rosemary M. Caron, Semra Aytur, Haylee Foster

**Affiliations:** ^1^Department of Health Management and Policy, University of New Hampshire, Durham, NH, United States; ^2^Department of Biological Sciences, University of New Hampshire, Durham, NH, United States

**Keywords:** public health, public health education, resilience, community health worker, public health workforce

## Abstract

*The Educated Citizen and Public Health* initiative promotes that an understanding of public health issues is a principal component of an educated population and is necessary to develop social responsibility and promote civic dialog. This initiative supports the Institute of Medicine’s (now the National Academy of Medicine) recommendation that “all undergraduates should have access to education in public health.” The purpose of our work is to examine the extent to which 2- and 4-year U.S. state colleges and universities offer and/or require a public health course. Select indicators identified include the presence and type of public health curriculum, public health course requirement, presence of public health graduate program offering, pathways to public health, Community Health Worker training, as well as demographic information for each institution. An analysis was also conducted for the historically Black colleges and universities (HBCUs), and the same select indicators were examined. The data suggest that there is an imperative need for a public health curriculum across the nation’s collegiate institutions with 26% of 4-year state institutions lacking a full undergraduate public health curriculum; 54% of 2-year colleges not offering a pathway to public health education; and 74% of HBCUs not offering a public health course or degree. In the age of COVID-19, syndemics, and considering the post-pandemic phase, we argue that expanding public health literacy at the associate and baccalaureate level can help prepare an educated citizenry who is both public health literate and one that can demonstrate resilience in the face of public health challenges.

## Introduction

The Institute of Medicine (IOM; today, known as the National Academy of Medicine) describes the mission of public health as “the fulfillment of society’s interest in assuring the conditions in which people can be healthy” (1). Although several decades old, this definition has withstood the test of numerous public health challenges and continues to succinctly present the overarching goal of an instrumental health field necessary to not only prevent disease, promote health, but to also protect the health of populations. The goal of public health is to achieve equity by utilizing a framework that promotes and protects health for all people in all communities. This goal is achieved by working to remove systemic and structural barriers (e.g., poverty, racism, ableism, and gender discrimination) that have contributed to health inequities and by promoting policies at the individual, community, and systems level that are aimed at achieving optimal health for all (2). Taken together, the core functions of public health and their related essential public health services contribute to the development of resilient communities which are connected socially and have accessible healthcare and public health systems able to withstand adversity and promote recovery (3). Resilient communities promote the social, behavioral, and physical health of residents to be able to respond to daily, as well as extreme challenges (3).

To emphasize the importance of public health education in contributing to healthy, resilient populations, the IOM promoted a call to action that “all undergraduates should have access to public health education” (4). This proposal was disseminated as an effort to advocate that an understanding of public health issues is an essential component of an educated public and is necessary to develop social responsibility while advocating for civic dialog. This endeavor is supported by the *Educated Citizen and Public Health* (5) initiative developed by the Association of American Colleges and Universities (AAC&U), the Association for Prevention Teaching and Research, and the Council on Colleges of Arts and Sciences. This effort highlights the urgency of learning basic public health principles for an educated citizenry to be responsive and resilient when faced with public health challenges.

The AAC&U advocates for all collegiate institutions to offer “broad knowledge and transferable skills, and a strong sense of values, ethics, and civic engagement” (6). In order to prepare an educated citizenry, a liberal education that provides adults with important life skills of analytical thinking, effective communication, and ethical judgment is essential (6). A public health education can contribute to this important initiative through its interdisciplinary nature. A basic knowledge of public health principles will aid in the AAC&U’s goal by contributing to an educated citizenry and workforce with the tools necessary to prepare for and respond to any number of public health emergencies, including the COVID-19 pandemic and ongoing syndemic threats such as food insecurity, structural racism, climate change, the opioid crisis, poor mental health, and homelessness. Thus, public health education can provide this knowledge to students in associate-level and baccalaureate-level academic institutions. A positive outcome of the COVID-19 pandemic has been the increased interest among college students in public health and the increased growth of undergraduate public health degree programs nationally that could benefit the predicted shortfall in the public health workforce (7). The purpose of our work is to examine the extent to which 2- and 4-year U.S. colleges and universities offer and/or require a public health course or content in their general education or major/minor curricula to contribute to the preparation of an educated and resilient citizenry thus fulfilling the IOM’s call to action.

## Methods

A qualitative analysis of the websites of 50 select academic U.S. state universities and 50 select U.S. community colleges was conducted. A state university in the U.S. is typically a public, collegiate institution that receives funding from the respective state’s government. These schools are traditionally attended by students for 4 years and the successful completion of degree requirements results in a Bachelor’s degree. In contrast, a community college provides a 2-year education with professional-focused courses and the successful completion of degree requirements results in an Associate’s degree. The selection of the state university/college reviewed was determined based on student enrollment and the flagship status of the school (flagship status refers to the academic institution that is the most well-known, or was established early from an historical perspective) within that state. The community colleges with the largest student population were selected for review. For the 107 historically black colleges and universities (HBCUs), a representative school was chosen for each state based on the student body size or flagship status for that given school. Not all states have an HBCU and therefore this did not pertain to all 50 states for which academic institutions were reviewed.

Select indicators were examined from each of the schools’ respective websites that were reviewed between February 2021 and August 2022. Select indicators were transcribed into an Excel™ spreadsheet and included the presence of a public health curriculum resulting in an Associate or Bachelor’s degree, type of curriculum (i.e., public health minor (defined as a secondary declared discipline or field of study during one’s undergraduate education); concentration (defined as a focused area of study within one’s major area of study); select courses, Master’s degree, Doctorate degree, Joint Graduate degree), public health courses fulfilling a general education course requirement, pathways to public health coursework, and whether Community Health Worker training was offered. The demographic information collected included student population size, state of school, and whether or not the institution is classified as a public and/or a land-grant institution (defined as an academic institution designated by a state or Congress to receive federal funding under the Morrill Acts of 1862 for agriculture education) (8).

A content analysis approach was used to provide “structure in a large amount of textual data through a systematic process of interpretation” (9). Specifically, a manifest content analysis was conducted as this approach focuses on data that is readily observable to the researcher and coder. The data were double-coded by two of the authors to address reliability. Frequency counts were used to identify specific categories and this approach reflects a surface-level type of analysis (9).

## Results

### Four-year state academic institutions

The average student body size for the 4-year state academic institutions reviewed was 28,744 and half of the 4-year institutions were classified as public and the other half were classified as public land-grant institutions. The following six themes were identified: public health curriculum resulting in a Bachelor degree; public health minor; public health concentration; health-related concentrations; required public health course in general education curriculum; and graduate public health education. Seventy-four percent of all 4-year state academic institutions examined offered a Bachelor of Arts or Bachelor of Science degree in public health thus a full curriculum is available. Twenty percent of the 4-year schools examined do not offer any public health educational programming within their academic institution. Of those 4-year institutions offering public health Bachelor’s degrees, 6% offered a public health minor or a public health concentration; 10% offered a health-related concentration (defined as a focused area of study within one’s major area of study in a health field that enables the student to address health needs from an individual, family, and/or community perspective). Further, none of the 4-year state academic institutions examined required a public health course as contributing to their general education requirement, yet more than half (54%) could potentially do so based on the course information reviewed. Public health education was more prevalent at the graduate level where 87.5% of state academic institutions offered a Master’s degree; 26% offered a Doctorate degree; and 16% of institutions offered a joint graduate degree with public health. With respect to the HBCUs examined, 26% offered a public health baccalaureate degree or a public health course.

### Two-year community colleges

The average student body size for the 2-year community colleges reviewed was 18,913 and all of the 2-year community colleges reviewed were public institutions. The following four themes were identified: public health curriculum resulting in an Associate’s degree; public health courses; courses related to public health; and a health-related degree. Ten percent of 2-year community colleges examined offer an Associate’s degree in public health and 20% offer a degree closely associated to public health, for example, health and wellness, community health, health informatics, and health administration. Of the community colleges examined, 17.5% offer courses related to public health that do not contribute to a certificate or degree. Table 1 summarizes the results from the analysis of 2- and 4-year academic institutions.

**Table 1:** Public health education at select 2- and 4-year U.S. academic institutions.

Type of U.S. academic institution	Public health major, minor, concentration, graduate degree	Public health courses required as part of the general education curriculum	Introductory-level courses offered in public health, epidemiology, global health
Four-year state colleges and universities	BA/BS in public health offered—74%	0% require a public health course as part of the general education curriculum	84% offer an introductory course in public health, epidemiology, and/or global health
	Public health minor/concentration—6%		
	No public health programming—20%		
	Health-related concentration—10%		
	Master’s—87.5%		
	Doctorate—26%		
Two-year community colleges	Associate’s degree in public health offered—10%	0% require a public health course as part of the general education curriculum	30% offer an introductory course in public health, epidemiology, and/or global health
	Health-related degree—20%		

### Public health education pathways

In support of the Institute of Medicine US (IOM)’s (4) call that all undergraduate students should have access to education in public health, 84% of 4-year state academic institutions offered an introductory-level course in public health, epidemiology, or global health, whereas 30% of community colleges offered such introductory courses. When considering the pathway of public health education options, 66% of 4-year state academic institutions provide a trajectory from a Bachelor’s degree to a Master’s degree; whereas 46% of 2-year community colleges offer a similar trajectory starting with the Associate’s degree. We further examined a Community Health Worker (CHW) pathway that could contribute to the public health workforce and found that 26% of 4-year state academic institutions offer training or certification for CHW compared to 46% of 2-year community colleges.

**Figure 1:**
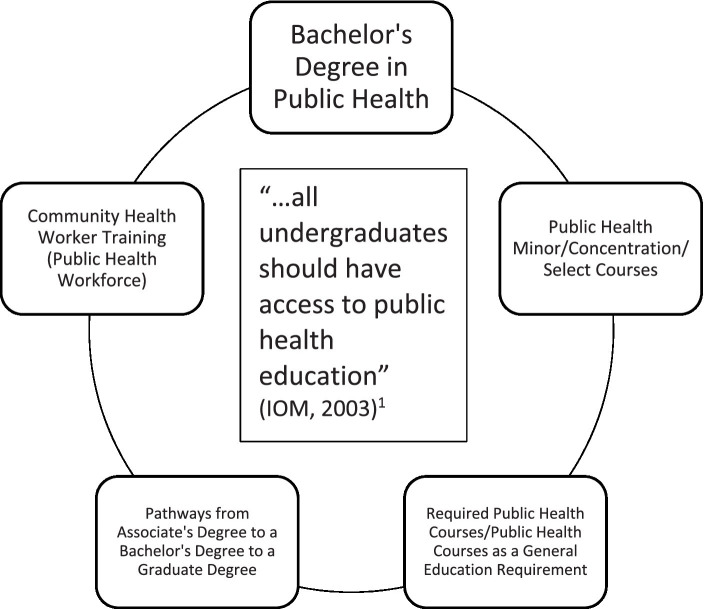
Types of public health education at select 2- and 4-year U.S. academic institutions (4).

Figure 1 represents the types of public health education at select 2- and 4-year U.S. academic institutions. Appendix provides a listing of the 2- and 4-year U.S. academic institutions reviewed.

It is important to note that the analysis of public health content and potential skill development resulting from a public health course or curriculum is limited to the material posted on each institution’s respective website. Based on the review of available course descriptions, the content for select public health courses does not present with much variation among the academic institutions reviewed. However, the community college system was more active in the offering of skill development for the Community Health Worker compared to the 4-year academic institutions which is expected as the mission of community colleges is to provide on-the-job training compared to 4-year institutions.

## Discussion

The COVID-19 pandemic has highlighted numerous public health challenges including, but not limited to, the understanding of the transmission and evolution of a novel virus, a disproportionate burden of morbidity and mortality on at-risk populations such as immigrants and refugees and those experiencing the effects of systemic racism, ongoing syndemics, increasing levels of misinformation, and an underestimation of health literacy as a public health problem globally (10–15). Select factors that contributed to the resilience of communities during the COVID-19 pandemic include “local knowledge, social networks, and communication,” as well as the ability to adapt one’s livelihood in response to the economic and environmental constraints imposed during the pandemic; innovation; and the sharing of collective learning as important for community resilience (16). Further, the integration among the public health, healthcare, and social service sectors can promote the protection of the population’s health and the efficient use of resources (17, 18). In accordance with the IOM’s call to action, we contend that the U.S. population could have been more resilient to this significant public health challenge if public health education had been more widely available at the 2- and 4-year post-secondary level, along with expanded public health infrastructure across multiple sectors. Although three-quarters of the 4-year U.S. state academic institutions offer a full curriculum in public health resulting in a Bachelor’s degree, recommendations on how to expand the public health education of a college-aged population that will be entering the workforce upon graduation could include requiring a public health course, even an introductory-level course, to fulfill a general education requirement at the 2- and 4-year institutional level for all students; promote 3 + 2 or 4 + 1 MPH Programs; and encourage collaboration among faculty from different disciplines to demonstrate the interdisciplinarity required to address public health challenges and achieve the public health mission. There is room for growth in developing minor degrees in public health, as well as concentration areas to complement one’s major area of study. These recommendations could contribute to not only educating the population in basic public health principles but also help to alleviate the public health workforce shortage by introducing young adults to the field of public health. It is estimated that in the post-COVID-19 era, the local and state governmental public health workforce in the U.S. requires 80,000 full-time employees to deliver foundational public health services (19).

The Community College is addressing the public health workforce shortage by offering certificates and 2-year degrees for Community Health Workers who are frontline workers in their respective communities fulfilling the mission of public health by working to reduce health disparities, providing access to care, and increasing screening (20). We note that if Community Colleges offer a CHW program without a feasible educational pathway for advancement, this could create a disparity where the CHW is not able to progress in wage earnings.

There has been tremendous growth in the development of accredited public health education with 26 standalone baccalaureate programs, 152 graduate programs in public health, and 67 schools of public health (21). The expansion of accredited public health educational programs provides the opportunity to not only contribute to building the public health workforce but to also introduce students at these respective institutions to the importance of public health in their everyday lives. The COVID-19 pandemic also contributed to an increase in student applications for graduate public health programs and schools as the importance of public health to assuring those conditions in which populations can be healthy were challenged and exposed by pre-existing multi-factorial public health issues, for example, violence, racism, social, and planetary determinants of health (22, 23). Further, students graduating with an undergraduate degree in public health increased by more than 1,100% between 2020 and 2021 which could also contribute to easing the public health workforce shortage (7).

The work presented herein possesses several limitations worthy of note. First, data collection was limited by public access to the website of select 2- and 4-year academic institutions, as well as the accuracy of the data that was presented on the websites examined. Also, many states have more than one state-based academic institution so the decision to analyze data from the selected academic institution could have resulted in incomplete information for a given state. Furthermore, this work presents a snapshot of the access to public health education for college students at select 2- and 4-year institutions, thus providing a descriptive overview of the availability of public health education at the time the study was conducted. Lastly, our findings serve as a starting point for further research and are not generalizable to all 2- and 4-year academic institutions in the United States.

In conclusion, the findings of this work indicate that there is room for improvement in fulfilling the Institute of Medicine’s (IOM) (4) call to action that “all undergraduates should have access to public health education.” This initiative has also been echoed internationally where the awareness of “core public health principles and concepts” and potential public health career options for those undergraduate students studying in the health professions or not should be made aware (24). Expanding public health education across 2- and 4-year academic institutions could allow for the development of more transdisciplinary courses/majors/minors (e.g., courses on the built environment involving faculty from public health, civil engineering, economics, and public administration) among faculty. Exposure to public health principles in one’s college experience could encourage life-long learning, a commitment to social responsibility, increased public health literacy, and resilience toward daily and substantial climate and anthropogenic challenges. Our work describes the current landscape of access to public health education at select 2- and 4-year academic institutions. Future work in this area includes examining the following questions: to what extent should public health education be introduced at the primary or secondary school levels? Does requiring public health education at the college level contribute to a more resilient population that is better prepared to manage the next public health crisis? What are the skills and expected competencies of one with a Bachelor’s degree in public health compared to an MPH degree? What are the primary job responsibilities for each level of preparedness? Which public health concepts should be standardized across states? What other pathways could contribute to the public health workforce, in addition to preparing CHWs? Further, to expand health education that utilizes a health promotion and disease prevention approach to a younger population of students, *Healthy People 2030* calls for an “Increase [in] the proportion of secondary schools that require students to take at least 2 health education courses between grades 6 and 12 (25).” What progress are we making on this national objective? Lastly, public health has historically been underfunded and although the CDC awarded local and state health departments more than $3 billion to build up infrastructure and expand the public health workforce (26), how will the funding that is needed to assure an adequate public health system be sustained? There is no shortage of public health challenges and reinforcing our commitment to the IOM’s call to action could help to support the public health mission for families, communities, and future generations.

## Data availability statement

The original contributions presented in the study are included in the article/Supplementary material, further inquiries can be directed to the corresponding author.

## Author contributions

RC proposed the topic for analysis/discussion and took the lead in writing the article. RC and SA co-developed the manuscript outline. HF conducted the analysis. All authors contributed to the article and approved the submitted version.

## Conflict of interest

The authors declare that the research was conducted in the absence of any commercial or financial relationships that could be construed as a potential conflict of interest.

## Publisher’s note

All claims expressed in this article are solely those of the authors and do not necessarily represent those of their affiliated organizations, or those of the publisher, the editors and the reviewers. Any product that may be evaluated in this article, or claim that may be made by its manufacturer, is not guaranteed or endorsed by the publisher.

## Supplementary material

The Supplementary material for this article can be found online at: https://www.frontiersin.org/articles/10.3389/fpubh.2023.1185845/full#supplementary-material
