# Supplementary material for: The Institute of Medicine’s call to action revisited: assuring access to public health education for U.S. college students
Source: Front Public Health. 2023 Apr 27;11:1185845. doi: 10.3389/fpubh.2023.1185845 (PMC10173863; doi:10.3389/fpubh.2023.1185845)
Supplement: Supplementary file 1 [file Table_1.DOCX]

**Appendix.** Select Two-, Four-Year, and HBCUs reviewed for accessible public health education.

**Four-Year Select U.S. State Universities**

- University of Alabama: <https://www.ua.edu/>
- University of Alaska Anchorage: <https://www.uaa.alaska.edu/>
- Arizona State University: <https://www.asu.edu/>
- Arkansas State University: <https://www.astate.edu/>
- California State University Northridge: <https://w2.csun.edu/>
- Colorado State University: <https://www.colostate.edu/>
- Central Connecticut State University: <https://www2.ccsu.edu/>
- University of Delaware: <https://www.udel.edu/>
- Florida State University: <https://www.fsu.edu/>
- Georgia State University: <https://www.gsu.edu/>
- University of Hawaii Manoa: <https://manoa.hawaii.edu/>
- Idaho State University: <https://www.isu.edu/>
- Illinois State University: <https://illinoisstate.edu/>
- Indiana State University: <https://www.indstate.edu/>
- Iowa State University: <https://www.iastate.edu/>
- Kansas State University: <https://www.k-state.edu/>
- University of Kentucky: <https://www.uky.edu/>
- Louisiana State University: <https://www.lsu.edu/>
- University of Maine Orono: <https://umaine.edu/>
- University of Maryland: <https://www.umd.edu/>
- University of Massachusetts Amherst: <https://www.umass.edu/>
- Michigan State University: <https://msu.edu/>
- Minnesota State University Moorhead: <https://www.mnstate.edu/>
- Mississippi State University: <https://www.msstate.edu/>
- Missouri State University: <https://www.missouristate.edu/>
- Montana State University: <https://www.montana.edu/>
- University of Nebraska Lincoln: <https://www.unl.edu/>
- University of Nevada Reno: <https://www.unr.edu/>
- University of New Hampshire: <https://www.unh.edu/>
- Rutgers University: <https://www.rutgers.edu/>
- New Mexico State University: <https://www.nmsu.edu/>
- University of Albany: <https://www.albany.edu/>
- North Carolina State University: <https://www.ncsu.edu/>
- North Dakota State University: <https://www.ndsu.edu/>
- Ohio State University: <https://www.osu.edu/>
- Oklahoma State University: <https://cas.okstate.edu/>
- Oregon State University: <https://oregonstate.edu/>
- Penn State University: <https://www.psu.edu/>
- University of Rhode Island: <https://www.uri.edu/>
- University of South Carolina: <https://sc.edu/>
- South Dakota University: <https://www.sdstate.edu/>
- University of Tennessee: <https://www.utk.edu/>
- Texas State University: <https://www.txst.edu/>
- Utah State University: <https://www.usu.edu/>
- University of Vermont: <https://www.uvm.edu/>
- University of Virginia: <https://www.virginia.edu/>
- Washington State University: <https://wsu.edu/>
- West Virginia University: <https://www.wvu.edu/>
- University of Wisconsin Milwaukee: <https://uwm.edu/>
- University of Wyoming: <http://www.uwyo.edu/>

**Two-Year Select U.S. Community Colleges**

- John C Calhoun State Community College: <https://calhoun.edu/>
- Prince William Sound Community College: <https://pwsc.alaska.edu/>
- Mesa Community College: <https://www.mesacc.edu/>
- North West Arkansas Community College: <https://www.nwacc.edu/>
- East Los Angeles Community College: <https://www.elac.edu/>
- Front Range Community College: <https://www.frontrange.edu/>
- Gateway Community College: <https://gatewayct.edu/>
- Delaware Technical Community College: <https://www.dtcc.edu/>
- Miami Dade Community College: <https://www.mdc.edu/>
- Perimeter College: <https://perimeter.gsu.edu/>
- Kapiolani Community College: <https://www.kapiolani.hawaii.edu/>
- College of Western Idaho: <https://cwi.edu/>
- College of DuPage: <https://cod.edu/>
- Ivy Tech Community College: <https://www.ivytech.edu/>
- Kirkwood Community College: <https://www.kirkwood.edu/>
- Johnson County Community College: <https://www.jccc.edu/>
- Jefferson Community and Technical College: <https://jefferson.kctcs.edu/>
- Delgado Community College: <https://www.dcc.edu/>
- Southern Maine Community College: <https://www.smccme.edu/>
- Montgomery College: <https://www.montgomerycollege.edu/>
- Bunker Hill Community College: <https://www.bhcc.edu/>
- Macomb Community College: <https://www.macomb.edu/>
- Normandale Community College: <https://www.normandale.edu/>
- Hinds Community College: <https://www.hindscc.edu/>
- Saint Louis Community College: <https://stlcc.edu/>
- Flathead Valley Community College: <https://www.fvcc.edu/>
- Metropolitan Community College: <https://www.mccneb.edu/>
- College of Southern Nevada: <https://www.csn.edu/>
- NHTI Concords Community College: <https://www.nhti.edu/>
- CUNY Borough of Manhattan Community College: <https://www.bmcc.cuny.edu/>
- Central New Mexico Community College: <https://www.cnm.edu/>
- Bergen Community College: <https://bergen.edu/>
- Wake Technical Community College: <https://www.waketech.edu/>
- Bismarck State College: <https://bismarckstate.edu/>
- Cuyahoga Community College: <https://www.tri-c.edu/>
- Oklahoma City Community College: <https://www.occc.edu/>
- Portland Community College: <https://www.pcc.edu/>
- Community College of Rhode Island: <https://www.ccri.edu/>
- Harrisburg Area Community College: <https://www.hacc.edu/>
- Trident Technical College: <https://www.tridenttech.edu/>
- Lake Area Technical College: <https://www.lakeareatech.edu/>
- Pellissippi State Community College: <https://www.pstcc.edu/>
- Lone Star College: <https://www.lonestar.edu/>
- Salt Lake Community College: <http://www.slcc.edu/>
- Community College of Vermont: <https://ccv.edu/>
- Northern Virginia Community College: <https://www.nvcc.edu/>
- Bellevue Community College: <https://www.bellevuecollege.edu/>
- Blue Ridge Community and Technical College: <https://www.blueridgectc.edu/>
- Milwaukee Area Technical College: <https://www.matc.edu/index.html>
- Casper College: <https://www.caspercollege.edu/>

**Historically Black Colleges and Universities (HBCUs)**

- Alabama State University: <https://www.alasu.edu/>
- University of Arkansas at Pine Bluff: <http://www.uapb.edu/>
- Charles R. Drew University of Medicine and Science: <https://www.cdrewu.edu/>
- Delaware State University: <https://www.desu.edu/>
- Florida A&M University: <https://www.famu.edu/>
- Albany State University: <https://www.asurams.edu/>
- Kentucky State University: <https://www.kysu.edu/>
- Southern University of New Orleans: <https://www.suno.edu/>
- University of Maryland Eastern Shore: <https://wwwcp.umes.edu/>
- Mississippi Valley State University: <https://www.mvsu.edu/>
- Harris-Stowe State University: <https://www.hssu.edu/>
- North Carolina A&T State University: <https://www.ncat.edu/>
- Central State University: <https://www.centralstate.edu/>
- Langston University: <https://www.langston.edu/>
- Lincoln University: <https://www.lincoln.edu/>
- South Carolina State University: <https://scsu.edu/>
- Tennessee State University: <https://www.tnstate.edu/>
- Texas Southern University: <http://www.tsu.edu/>
- Virginia State University: <https://www.vsu.edu/>
- West Virginia State University: <https://www.wvstateu.edu/>
